# (4*Z*)-4-Benzyl­idene-2-phenyl-1,3-oxazol-5(4*H*)-one

**DOI:** 10.1107/S1600536812011579

**Published:** 2012-03-24

**Authors:** Abdullah M. Asiri, Hassan M. Faidallah, Tariq R. Sobahi, Seik Weng Ng, Edward R. T. Tiekink

**Affiliations:** a Chemistry Department, Faculty of Science, King Abdulaziz University, PO Box 80203, Jeddah, Saudi Arabia; b The Center of Excellence for Advanced Materials Research, King Abdulaziz University, Jeddah, PO Box 80203, Saudi Arabia; c Department of Chemistry, University of Malaya, 50603 Kuala Lumpur, Malaysia

## Abstract

In the title compound, C_17_H_13_NO_2_, the benzene ring is twisted slightly out of the plane of the oxazole ring to which it is attached [dihedral angle = 7.98 (8)°]. Similarly, there is a twist [dihedral angle = 5.50 (8)°] between the oxazole and phenyl rings that are linked *via* the C=C bond [1.348 (2) Å]; the conformation about the latter is *Z*. In the crystal, the presence of C—H⋯O, C—H⋯π and π–π inter­actions [centroid–centroid distance = 3.5259 (9) Å] link the mol­ecules into a three-dimensional architecture.

## Related literature

For background to the biological activity of oxazolone derivatives, see: Fidanza & Dernoeden (1996 ▶); Khan *et al.* (2006 ▶); Puig *et al.* (2000 ▶) For the synthesis, see: Mariappan *et al.* (2011 ▶).
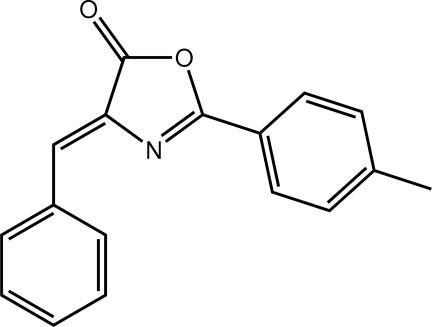


## Experimental

### 

#### Crystal data

C_17_H_13_NO_2_
*M* *_r_* = 263.28
Orthorhombic, 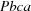

*a* = 12.0827 (6) Å
*b* = 7.7848 (3) Å
*c* = 27.6527 (16) Å
*V* = 2601.1 (2) Å^3^
*Z* = 8
Mo *K*α radiation
μ = 0.09 mm^−1^
*T* = 100 K
0.30 × 0.25 × 0.20 mm

#### Data collection

Agilent SuperNova Dual diffractometer with an Atlas detector
Absorption correction: multi-scan (*CrysAlis PRO*; Agilent, 2011 ▶) *T*_min_ = 0.974, *T*_max_ = 0.983
7121 measured reflections
2990 independent reflections
2206 reflections with *I* > 2σ(*I*)
*R*_int_ = 0.033

#### Refinement

*R*[*F* ^2^ > 2σ(*F* ^2^)] = 0.044
*wR*(*F* ^2^) = 0.109
*S* = 1.03
2990 reflections
182 parameters
H-atom parameters constrained
Δρ_max_ = 0.22 e Å^−3^
Δρ_min_ = −0.24 e Å^−3^

### 

Data collection: *CrysAlis PRO* (Agilent, 2011 ▶); cell refinement: *CrysAlis PRO*; data reduction: *CrysAlis PRO*; program(s) used to solve structure: *SHELXS97* (Sheldrick, 2008 ▶); program(s) used to refine structure: *SHELXL97* (Sheldrick, 2008 ▶); molecular graphics: *ORTEP-3* (Farrugia, 1997 ▶) and *DIAMOND* (Brandenburg, 2006 ▶); software used to prepare material for publication: *publCIF* (Westrip, 2010 ▶).

## Supplementary Material

Crystal structure: contains datablock(s) global, I. DOI: 10.1107/S1600536812011579/hb6681sup1.cif

Structure factors: contains datablock(s) I. DOI: 10.1107/S1600536812011579/hb6681Isup2.hkl

Supplementary material file. DOI: 10.1107/S1600536812011579/hb6681Isup3.cml

Additional supplementary materials:  crystallographic information; 3D view; checkCIF report

## Figures and Tables

**Table 1:** Hydrogen-bond geometry (Å, °) *Cg*1 is the centroid of the C5–C10 ring.

*D*—H⋯*A*	*D*—H	H⋯*A*	*D*⋯*A*	*D*—H⋯*A*
C7—H7⋯O2^i^	0.95	2.56	3.463 (2)	158
C6—H6⋯*Cg*1^ii^	0.95	2.93	3.8311 (17)	158
C9—H9⋯*Cg*1^iii^	0.95	2.92	3.6532 (17)	135

Symmetry codes: (i) 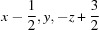; (ii) 
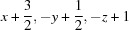
; (iii) 
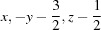
.

**Fig. 1.**
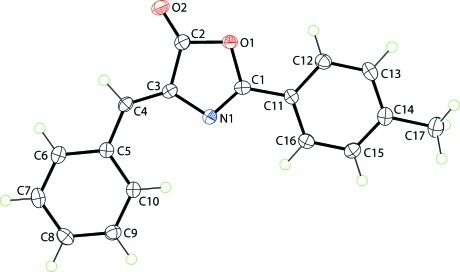
The molecular structure of (I) showing displacement ellipsoids at the 50% probability level.

**Fig. 2.**
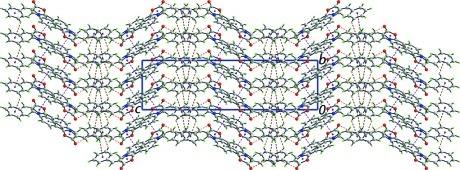
A view in projection down the a axis of the unit-cell contents of (I). The C—H···O, C—H···π and π—π interactions are shown as orange, brown and purple dashed lines, respectively.

## supplementary crystallographic information

### Comment

Several oxazolone derivatives (Fidanza & Dernoeden, 1996) have proved effective as insecticides, herbicides and fungicides that control brown patch (*Rhizoctonia solani* (Kühn)). Oxazol-5-ones are known to inhibit the activity of the tyrosinase enzyme with a maximum inhibition by the derivative which bears a cinnamoyl residue at the C-4 position (Khan *et al.*, 2006). Further, some 3,4-diaryloxazolones show inhibition of cyclooxygenase-2 (COX-2) and *in vivo* anti-inflammatory activity making them excellent candidates for the treatment of arthritis and hyperalgesia (Puig *et al.*, 2000). In this connection, the title compound, 4(*Z*)-2-phenyl-4-(phenylmethylidene)-4,5-dihydro-1,3-oxazol-5-one (I), was synthesized and characterized by X-ray crystallography.

In (I), Fig. 1, the oxazole ring is planar with a r.m.s. deviation for the fitted atoms of 0.007 Å. The pendent benzene ring is slightly twisted out of this plane and forms a dihedral angle of 7.98 (8)°; the N1—C1—C11—C12 torsion angle = -171.85 (15)°. The conformation about the C3═C4 bond [1.348 (2) Å] is *Z*. There is a slight twist in this region of the molecule so that the dihedral angle between the oxazol and phenyl rings is 5.50 (8)°; the C4—C5—C10—C9 torsion angle = 177.79 (14)°. The r.m.s. deviation of the 20 non-hydrogen atoms comprising (I) = 0.131 Å with the maximum deviations being 0.258 (1) Å for the C16 atom and -0.224 (2) Å for the C13 atom.

The crystal packing is sustained by C—H···O and C—H···π interactions, Table 1, as well as π—π interactions occurring between the oxazole and benzene rings [ring centroid···ring centroid distance = 3.5259 (9) Å for symmetry operation 1 - *x*, 1 - *y*, 1 - *z*]. Globally, molecules assemble into undulating layers that stack along the *b* axis, Fig. 2.

### Experimental

4-Methoxybenzoylglycine was prepared in accord with the literature procedure (Mariappan *et al.*, 2011). A mixture of 4-methoxybenzoylglycine (2.1 g, 0.01 mmol), benzaldehyde (1.1 g, 0.02 mmol), anhydrous sodium acetate (0.8 g, 0.01 mmol) and acetic anhydride (4.0 g, 0.04 mmol) was refluxed for 1 h on a water bath with occasional stirring. The resulting mixture was left in a refrigerator overnight. The solid thus obtained was filtered, washed with cold water, dried in an hot-air oven at 333 K and recrystallized from ethanol as yellow polyhedra. Yield: 84%. *M*.pt: 470–471 K.

### Refinement

Carbon-bound H-atoms were placed in calculated positions [C—H = 0.95 to 0.98 Å, *U*_iso_(H) = 1.2 to 1.5*U*_eq_(C)] and were included in the refinement in the riding model approximation.

### Crystal data

**Table:**

C_17_H_13_NO_2_	*F*(000) = 1104
*M**_r_* = 263.28	*D*_x_ = 1.345 Mg m^−^^3^
Orthorhombic, *P**b**c**a*	Mo *K*α radiation, λ = 0.71073 Å
Hall symbol: -P 2ac 2ab	Cell parameters from 2443 reflections
*a* = 12.0827 (6) Å	θ = 2.6–27.5°
*b* = 7.7848 (3) Å	µ = 0.09 mm^−^^1^
*c* = 27.6527 (16) Å	*T* = 100 K
*V* = 2601.1 (2) Å^3^	Polyhedron, yellow
*Z* = 8	0.30 × 0.25 × 0.20 mm

### Data collection

**Table:**

Agilent SuperNova Dual diffractometer with an Atlas detector	2990 independent reflections
Radiation source: SuperNova (Mo) X-ray Source	2206 reflections with *I* > 2σ(*I*)
Mirror monochromator	*R*_int_ = 0.033
Detector resolution: 10.4041 pixels mm^-1^	θ_max_ = 27.6°, θ_min_ = 3.0°
ω scan	*h* = −15→9
Absorption correction: multi-scan (*CrysAlis PRO*; Agilent, 2011)	*k* = −7→10
*T*_min_ = 0.974, *T*_max_ = 0.983	*l* = −36→20
7121 measured reflections	

### Refinement

**Table:**

Refinement on *F*^2^	Primary atom site location: structure-invariant direct methods
Least-squares matrix: full	Secondary atom site location: difference Fourier map
*R*[*F*^2^ > 2σ(*F*^2^)] = 0.044	Hydrogen site location: inferred from neighbouring sites
*wR*(*F*^2^) = 0.109	H-atom parameters constrained
*S* = 1.03	*w* = 1/[σ^2^(*F*_o_^2^) + (0.043*P*)^2^ + 0.6059*P*] where *P* = (*F*_o_^2^ + 2*F*_c_^2^)/3
2990 reflections	(Δ/σ)_max_ = 0.001
182 parameters	Δρ_max_ = 0.22 e Å^−^^3^
0 restraints	Δρ_min_ = −0.24 e Å^−^^3^

### Special details

**Table:**

Geometry. All e.s.d.'s (except the e.s.d. in the dihedral angle between two l.s. planes) are estimated using the full covariance matrix. The cell e.s.d.'s are taken into account individually in the estimation of e.s.d.'s in distances, angles and torsion angles; correlations between e.s.d.'s in cell parameters are only used when they are defined by crystal symmetry. An approximate (isotropic) treatment of cell e.s.d.'s is used for estimating e.s.d.'s involving l.s. planes.

### Fractional atomic coordinates and isotropic or equivalent isotropic displacement parameters (Å^2^)

**Table:**

	*x*	*y*	*z*	*U*_iso_*/*U*_eq_	
O1	0.70006 (8)	0.51990 (13)	0.55832 (4)	0.0199 (3)	
O2	0.76383 (9)	0.68781 (14)	0.61933 (4)	0.0270 (3)	
N1	0.53733 (10)	0.42459 (15)	0.58986 (4)	0.0174 (3)	
C1	0.60411 (12)	0.42309 (18)	0.55339 (6)	0.0176 (3)	
C2	0.69316 (13)	0.59344 (19)	0.60408 (6)	0.0197 (3)	
C3	0.58820 (12)	0.52949 (18)	0.62455 (6)	0.0176 (3)	
C4	0.55689 (12)	0.56871 (18)	0.67001 (6)	0.0186 (3)	
H4	0.6050	0.6445	0.6868	0.022*	
C5	0.46027 (12)	0.51218 (18)	0.69718 (6)	0.0176 (3)	
C6	0.44944 (13)	0.5668 (2)	0.74539 (6)	0.0220 (4)	
H6	0.5037	0.6409	0.7590	0.026*	
C7	0.36046 (14)	0.51376 (19)	0.77344 (6)	0.0238 (4)	
H7	0.3538	0.5520	0.8059	0.029*	
C8	0.28135 (14)	0.40496 (19)	0.75395 (6)	0.0233 (4)	
H8	0.2205	0.3683	0.7731	0.028*	
C9	0.29095 (13)	0.3493 (2)	0.70625 (6)	0.0226 (4)	
H9	0.2368	0.2741	0.6931	0.027*	
C10	0.37888 (12)	0.40289 (19)	0.67789 (6)	0.0200 (3)	
H10	0.3842	0.3656	0.6453	0.024*	
C11	0.59187 (12)	0.32991 (18)	0.50822 (5)	0.0169 (3)	
C12	0.67737 (13)	0.3232 (2)	0.47430 (6)	0.0221 (4)	
H12	0.7441	0.3847	0.4799	0.027*	
C13	0.66513 (13)	0.2269 (2)	0.43248 (6)	0.0249 (4)	
H13	0.7241	0.2222	0.4098	0.030*	
C14	0.56739 (13)	0.13662 (19)	0.42313 (6)	0.0216 (3)	
C15	0.48133 (13)	0.14847 (19)	0.45658 (6)	0.0211 (3)	
H15	0.4136	0.0906	0.4504	0.025*	
C16	0.49287 (12)	0.24313 (18)	0.49867 (6)	0.0195 (3)	
H16	0.4335	0.2491	0.5211	0.023*	
C17	0.55537 (15)	0.0289 (2)	0.37818 (6)	0.0292 (4)	
H17A	0.5060	−0.0682	0.3848	0.044*	
H17B	0.6282	−0.0144	0.3684	0.044*	
H17C	0.5241	0.0990	0.3521	0.044*	

### Atomic displacement parameters (Å^2^)

**Table:**

	*U*^11^	*U*^22^	*U*^33^	*U*^12^	*U*^13^	*U*^23^
O1	0.0168 (5)	0.0235 (5)	0.0194 (6)	−0.0029 (4)	−0.0007 (4)	0.0022 (5)
O2	0.0260 (6)	0.0308 (6)	0.0242 (6)	−0.0105 (5)	−0.0044 (5)	0.0035 (5)
N1	0.0182 (6)	0.0171 (6)	0.0168 (7)	0.0012 (5)	−0.0021 (5)	0.0014 (5)
C1	0.0160 (7)	0.0164 (7)	0.0204 (8)	0.0007 (6)	−0.0025 (6)	0.0053 (6)
C2	0.0216 (8)	0.0197 (7)	0.0176 (8)	−0.0005 (7)	−0.0043 (6)	0.0046 (6)
C3	0.0181 (7)	0.0154 (7)	0.0193 (8)	−0.0001 (6)	−0.0042 (6)	0.0030 (6)
C4	0.0189 (7)	0.0167 (7)	0.0201 (8)	0.0004 (6)	−0.0050 (6)	0.0000 (6)
C5	0.0197 (7)	0.0149 (7)	0.0182 (8)	0.0029 (6)	−0.0014 (6)	0.0019 (6)
C6	0.0249 (8)	0.0205 (7)	0.0207 (8)	0.0017 (7)	−0.0030 (7)	−0.0012 (7)
C7	0.0315 (9)	0.0230 (8)	0.0167 (8)	0.0075 (7)	0.0003 (7)	0.0002 (7)
C8	0.0242 (8)	0.0223 (8)	0.0232 (9)	0.0035 (7)	0.0051 (7)	0.0045 (7)
C9	0.0225 (8)	0.0201 (8)	0.0251 (9)	−0.0020 (7)	0.0002 (7)	−0.0004 (7)
C10	0.0214 (8)	0.0202 (7)	0.0184 (8)	0.0012 (6)	−0.0004 (6)	−0.0010 (6)
C11	0.0181 (7)	0.0166 (7)	0.0159 (8)	0.0037 (6)	−0.0004 (6)	0.0032 (6)
C12	0.0192 (7)	0.0249 (8)	0.0222 (9)	0.0008 (7)	0.0001 (7)	0.0015 (7)
C13	0.0250 (8)	0.0293 (8)	0.0203 (9)	0.0052 (7)	0.0049 (7)	0.0001 (7)
C14	0.0294 (8)	0.0164 (7)	0.0189 (8)	0.0036 (7)	−0.0019 (7)	0.0030 (6)
C15	0.0230 (8)	0.0180 (7)	0.0225 (8)	−0.0012 (6)	−0.0030 (7)	0.0028 (6)
C16	0.0199 (8)	0.0185 (7)	0.0202 (8)	0.0013 (7)	0.0006 (6)	0.0044 (6)
C17	0.0386 (10)	0.0238 (8)	0.0253 (9)	0.0016 (8)	−0.0009 (8)	−0.0033 (7)

### Geometric parameters (Å, º)

**Table:**

O1—C2	1.3913 (19)	C9—C10	1.385 (2)
O1—C1	1.3895 (18)	C9—H9	0.9500
O2—C2	1.2028 (18)	C10—H10	0.9500
N1—C1	1.2915 (19)	C11—C12	1.396 (2)
N1—C3	1.4017 (19)	C11—C16	1.399 (2)
C1—C11	1.452 (2)	C12—C13	1.386 (2)
C2—C3	1.475 (2)	C12—H12	0.9500
C3—C4	1.348 (2)	C13—C14	1.399 (2)
C4—C5	1.456 (2)	C13—H13	0.9500
C4—H4	0.9500	C14—C15	1.395 (2)
C5—C10	1.406 (2)	C14—C17	1.507 (2)
C5—C6	1.405 (2)	C15—C16	1.385 (2)
C6—C7	1.388 (2)	C15—H15	0.9500
C6—H6	0.9500	C16—H16	0.9500
C7—C8	1.386 (2)	C17—H17A	0.9800
C7—H7	0.9500	C17—H17B	0.9800
C8—C9	1.393 (2)	C17—H17C	0.9800
C8—H8	0.9500		
			
C2—O1—C1	105.22 (11)	C8—C9—H9	119.8
C1—N1—C3	105.41 (12)	C9—C10—C5	120.29 (15)
N1—C1—O1	116.08 (13)	C9—C10—H10	119.9
N1—C1—C11	127.78 (14)	C5—C10—H10	119.9
O1—C1—C11	116.13 (13)	C12—C11—C16	119.18 (14)
O2—C2—O1	121.84 (14)	C12—C11—C1	121.42 (14)
O2—C2—C3	133.00 (15)	C16—C11—C1	119.39 (13)
O1—C2—C3	105.16 (12)	C13—C12—C11	120.13 (15)
C4—C3—N1	130.33 (14)	C13—C12—H12	119.9
C4—C3—C2	121.51 (14)	C11—C12—H12	119.9
N1—C3—C2	108.12 (13)	C12—C13—C14	121.08 (15)
C3—C4—C5	129.62 (14)	C12—C13—H13	119.5
C3—C4—H4	115.2	C14—C13—H13	119.5
C5—C4—H4	115.2	C15—C14—C13	118.27 (15)
C10—C5—C6	118.55 (14)	C15—C14—C17	120.80 (15)
C10—C5—C4	123.23 (14)	C13—C14—C17	120.93 (15)
C6—C5—C4	118.21 (14)	C16—C15—C14	121.17 (15)
C7—C6—C5	120.80 (15)	C16—C15—H15	119.4
C7—C6—H6	119.6	C14—C15—H15	119.4
C5—C6—H6	119.6	C15—C16—C11	120.13 (14)
C8—C7—C6	119.90 (15)	C15—C16—H16	119.9
C8—C7—H7	120.1	C11—C16—H16	119.9
C6—C7—H7	120.1	C14—C17—H17A	109.5
C7—C8—C9	120.05 (15)	C14—C17—H17B	109.5
C7—C8—H8	120.0	H17A—C17—H17B	109.5
C9—C8—H8	120.0	C14—C17—H17C	109.5
C10—C9—C8	120.41 (15)	H17A—C17—H17C	109.5
C10—C9—H9	119.8	H17B—C17—H17C	109.5
			
C3—N1—C1—O1	−0.47 (16)	C6—C7—C8—C9	−0.2 (2)
C3—N1—C1—C11	178.31 (14)	C7—C8—C9—C10	−0.4 (2)
C2—O1—C1—N1	−0.24 (16)	C8—C9—C10—C5	0.9 (2)
C2—O1—C1—C11	−179.16 (12)	C6—C5—C10—C9	−0.7 (2)
C1—O1—C2—O2	−179.03 (14)	C4—C5—C10—C9	177.79 (14)
C1—O1—C2—C3	0.80 (14)	N1—C1—C11—C12	−171.85 (15)
C1—N1—C3—C4	−176.66 (15)	O1—C1—C11—C12	6.9 (2)
C1—N1—C3—C2	0.95 (15)	N1—C1—C11—C16	7.3 (2)
O2—C2—C3—C4	−3.4 (3)	O1—C1—C11—C16	−173.88 (12)
O1—C2—C3—C4	176.76 (13)	C16—C11—C12—C13	−2.1 (2)
O2—C2—C3—N1	178.70 (16)	C1—C11—C12—C13	177.12 (14)
O1—C2—C3—N1	−1.10 (15)	C11—C12—C13—C14	0.7 (2)
N1—C3—C4—C5	0.6 (3)	C12—C13—C14—C15	1.2 (2)
C2—C3—C4—C5	−176.76 (14)	C12—C13—C14—C17	−178.58 (14)
C3—C4—C5—C10	−1.3 (2)	C13—C14—C15—C16	−1.7 (2)
C3—C4—C5—C6	177.19 (15)	C17—C14—C15—C16	178.08 (14)
C10—C5—C6—C7	0.1 (2)	C14—C15—C16—C11	0.3 (2)
C4—C5—C6—C7	−178.48 (14)	C12—C11—C16—C15	1.6 (2)
C5—C6—C7—C8	0.3 (2)	C1—C11—C16—C15	−177.63 (13)

### Hydrogen-bond geometry (Å, º)

Cg1 is the centroid of the C5–C10 ring.

**Table:**

*D*—H···*A*	*D*—H	H···*A*	*D*···*A*	*D*—H···*A*
C7—H7···O2^i^	0.95	2.56	3.463 (2)	158
C6—H6···*Cg*1^ii^	0.95	2.93	3.8311 (17)	158
C9—H9···*Cg*1^iii^	0.95	2.92	3.6532 (17)	135

Symmetry codes: (i) *x*−1/2, *y*, −*z*+3/2; (ii) *x*+3/2, −*y*+1/2, −*z*+1; (iii) *x*, −*y*−3/2, *z*−1/2.
