# (4*Z*)-4-Benzyl­idene-2-phenyl-1,3-oxazol-5(4*H*)-one. Corrigendum

**DOI:** 10.1107/S2056989015019271

**Published:** 2015-10-17

**Authors:** Abdullah M. Asiri, Hassan M. Faidallah, Tariq R. Sobahi, Seik Weng Ng, Edward R. T. Tiekink

**Affiliations:** a Chemistry Department, Faculty of Science, King Abdulaziz University, PO Box 80203, Jeddah, Saudi Arabia; b The Center of Excellence for Advanced Materials Research, King Abdulaziz University, Jeddah, PO Box 80203, Saudi Arabia; c Department of Chemistry, University of Malaya, 50603 Kuala Lumpur, Malaysia

## Abstract

In the paper by Asiri *et al.* [*Acta Cryst.* (2012), E**68**, o1154], the title and the chemical name of one of the reagents used in the synthesis are corrected.

## Abstract 2

Corrigendum to *Acta Cryst.* (2012), E**68**, o1154.

In the paper by Asiri *et al.* (2012) ▸, the chemical name given in the title should be ‘(4*Z*)-4-Benzyl­idene-2-(4-methylphenyl)-1,3-oxazol-5(4*H*)-one’. In the *Experimental* section of the *Supporting information*, the reagent used was ‘4-methyl­benz­oylglycine’ and not ‘4-methoxybenzoylglycine’ as stated.
